# *Staphylococcus* spp. in Salad Vegetables: Biodiversity, Antimicrobial Resistance, and First Identification of Methicillin-Resistant Strains in the United Arab Emirates Food Supply

**DOI:** 10.3390/foods13152439

**Published:** 2024-08-02

**Authors:** Ihab Habib, Glindya Bhagya Lakshmi, Mohamed-Yousif Ibrahim Mohamed, Akela Ghazawi, Mushtaq Khan, Rami H. Al-Rifai, Afra Abdalla, Febin Anes, Mohammed Elbediwi, Hazim O. Khalifa, Abiola Senok

**Affiliations:** 1 Department of Veterinary Medicine, College of Agriculture and Veterinary Medicine, United Arab Emirates University, Al Ain P.O. Box 15551, United Arab Emirates; glindya_l@uaeu.ac.ae (G.B.L.); mohamed-yousif-i@uaeu.ac.ae (M.-Y.I.M.); afra.abdalla@uaeu.ac.ae (A.A.); hfebin@uaeu.ac.ae (F.A.); hazimkhalifa@uaeu.ac.ae (H.O.K.); 2 ASPIRE Research Institute for Food Security in the Drylands (ARIFSID), United Arab Emirates University, Al Ain P.O. Box 15551, United Arab Emirates; 3 Department of Medical Microbiology and Immunology, College of Medicine and Health Sciences, United Arab Emirates University, Al Ain P.O. Box 15551, United Arab Emirates; akelag@uaeu.ac.ae (A.G.); mushtaq.khan@uaeu.ac.ae (M.K.); 4 Zayed Center for Health Sciences, United Arab Emirates University, Al Ain P.O. Box 15551, United Arab Emirates; rrifai@uaeu.ac.ae; 5 Institute of Public Health, College of Medicine and Health Sciences, United Arab Emirates University, Al Ain P.O. Box 15551, United Arab Emirates; 6 Evolutionary Biology, Institute for Biology, Freie Universität Berlin, 14195 Berlin, Germany; mohammed.elbediwi@fu-berlin.de; 7 Animal Health Research Institute, Agriculture Research Centre, Cairo 12619, Egypt; 8 Department of Pharmacology, Faculty of Veterinary Medicine, Kafrelsheikh University, Kafr El Sheikh 33516, Egypt; 9 College of Medicine, Mohammed Bin Rashid University of Medicine and Health Sciences, Dubai P.O. Box 50505, United Arab Emirates; abiola.senok@mbru.ac.ae; 10 School of Dentistry, Cardiff University, Cardiff CF14 4XY, UK

**Keywords:** fresh produce, United Arab Emirates, food safety, antimicrobial resistance, *Staphylococci*

## Abstract

Contamination of leafy greens with *Staphylococcus* spp. can occur at various supply chain stages, from farm to table. This study comprehensively analyzes the species diversity, antimicrobial resistance, and virulence factors of *Staphylococci* in salad vegetables from markets in the United Arab Emirates (UAE). A total of 343 salad items were sampled from three major cities in the UAE from May 2022 to February 2023 and tested for the presence of *Staphylococcus* spp. using standard culture-based methods. Species-level identification was achieved using matrix-assisted laser desorption ionization-time of flight mass spectrometry. Antimicrobial susceptibility testing was conducted using the VITEK-2 system with AST-P592 cards. Additionally, whole genome sequencing (WGS) of ten selected isolates was performed to characterize antimicrobial resistance determinants and toxin-related virulence factors. Nine *Staphylococcus* species were identified in 37.6% (129/343) of the tested salad items, with coagulase-negative *staphylococci* (CoNS) dominating (87.6% [113/129]) and *S. xylosus* being the most prevalent (89.4% [101/113]). *S. aureus* was found in 4.6% (14/343) of the salad samples, averaging 1.7 log10 CFU/g. One isolate was confirmed as methicillin-resistant *S. aureus*, harboring the *mecA* gene. It belonged to multi-locus sequence type ST-672 and spa type t384 and was isolated from imported fresh dill. Among the characterized *S. xylosus* (*n* = 45), 13.3% tested positive in the cefoxitin screen test, and 6.6% were non-susceptible to oxacillin. WGS analysis revealed that the cytolysin gene (*cylR2*) was the only toxin-associated factor found in *S. xylosus*, while a methicillin-sensitive *S. aureus* isolate harbored the Panton-Valentine Leukocidin (*LukSF*/*PVL*) gene. This research is the first to document the presence of methicillin-resistant *S. aureus* in the UAE food chain. Furthermore, *S. xylosus* (a coagulase-negative *staphylococcus* not commonly screened in food) has demonstrated phenotypic resistance to clinically relevant antimicrobials. This underscores the need for vigilant monitoring of antimicrobial resistance in bacterial contaminants, whether pathogenic or commensal, at the human-food interface.

## 1. Introduction

Leafy greens are an important part of a nutritious diet and are widely appreciated for their health benefits. Nonetheless, they are prone to contamination by various microorganisms, including *Staphylococcus* spp., which pose significant public health concerns [1,2]. These bacteria are widely present in various ecosystems, such as soil, water, animal hide, skin, and food. The coagulase-positive (CoPS) species *Staphylococcus aureus* (*S. aureus*) is particularly infamous for causing foodborne intoxication, with symptoms including nausea, vomiting, and diarrhea [3]. Additionally, this pathogenic bacterium can exhibit resistance to various antibiotics; notably, methicillin-resistant *S. aureus* (MRSA) strains—associated with communities, livestock, and healthcare settings—are increasingly being detected in food products and among workers in food processing sectors [3,4].

While coagulase-positive *S. aureus* often attracts significant attention, coagulase-negative *staphylococci* (CoNS) have also become increasingly noteworthy in recent years [2]. Species such as *Staphylococcus epidermidis*, *Staphylococcus haemolyticus*, and *Staphylococcus saprophyticus* are now recognized as causes of nosocomial infections in humans, while *Staphylococcus xylosus* (*S. xylosus*) has also been reported to cause infections in animals [2,5]. Furthermore, the spread of antibiotic resistance among CoNS, which is reported to harbor resistance genes, poses a potential health hazard [6]. This hazard arises from the possible transfer of resistance genes between *staphylococcal* species and the direct transmission of resistant pathogens to humans, primarily via foods that are consumed ready-to-eat or not receiving a bactericidal (e.g., heat/cooking) treatment [6,7]. These characteristics highlight the importance of evaluating the presence of both CoPS and CoNS in various food environments and items, including leafy greens.

Contamination of leafy greens with *Staphylococcus* spp. can occur at various stages of the supply chain, from farm to table. Poor hygiene practices during cultivation, harvesting, handling, and retailing can introduce these bacteria to the produce [1]. Studies have demonstrated that leafy greens, often consumed raw, are particularly susceptible to microbial contamination [8]. Surveys conducted globally, including in China and Europe, have documented the prevalence of *Staphylococcus* spp. in leafy greens, underscoring the need for ongoing monitoring and stringent control measures [9,10].

The rise of antimicrobial resistance (AMR) in *Staphylococcus* spp. is a growing global concern [2]. The misuse of antibiotics in agriculture and healthcare has accelerated the development of resistant strains. Methicillin-resistant *S. aureus* (MRSA) has become a significant cause of healthcare-associated infections globally and is now recognized as an emerging pathogen outside healthcare settings [4]. Similarly, CoNS have developed resistance mechanisms against multiple classes of antibiotics, including tetracycline, glycopeptides, and aminoglycosides [5]. In the UAE, where consumption of leafy greens is increasing due to heightened health awareness, understanding the prevalence and antimicrobial resistance patterns of *Staphylococcus* spp. in salad greens is crucial. Hence, this study aims to identify the presence of both CoPS and CoNS in fresh salad items sampled from UAE retail and to evaluate their antimicrobial resistance profiles. Furthermore, a subset of the recovered isolates was characterized using whole genome sequencing (WGS), and we report for the first time in the UAE the presence and genomic features of an MRSA (as well as methicillin-susceptible) *S. aureus* detected in leafy greens. This study provides evidence-based findings to enhance food safety protocols and inform public health policies towards a better hazard characterization of *Staphylococcus* spp. in the fresh produce supply chain.

## 2. Materials and Methods

### 2.1. Sampling and Study Setting

**Figure 1:**
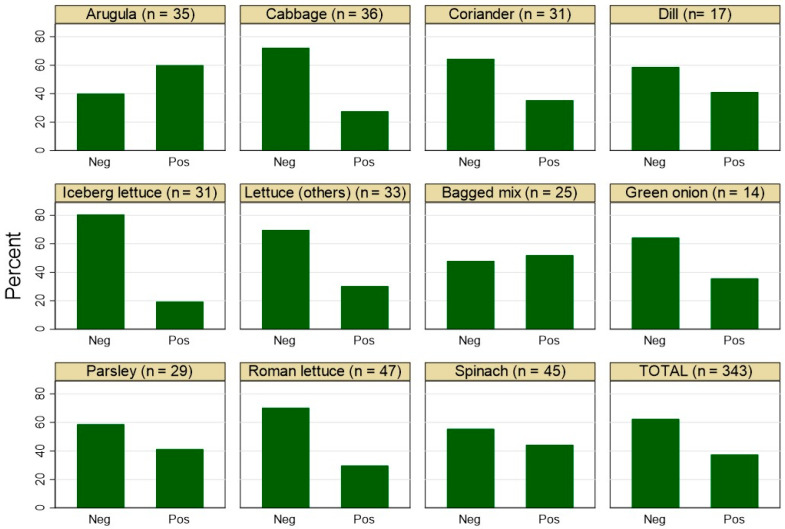
Proportion of positive (Pos) and negative (Neg) *Staphylococcus* spp. screened in different salad items (*n* = 343) sampled from markets in the United Arab Emirates.

A sampling frame was developed by collecting samples from fresh salad vegetable items from public vegetable markets and retail supermarkets in some of the major cities in the UAE (Abu Dhabi, Al Ain, and Dubai). The sample size, calculated for a 50% prevalence rate with a 90% confidence level and a 5% error margin, comprised 343 samples collected over the period from May 2022 to February 2023. The collected samples included a variety of salad items such as arugula, romaine lettuce, spinach, other lettuce varieties, parsley, cabbage, coriander, iceberg lettuce, mixed salad packs, dill, and onion leaves, with their distribution presented in Figure 1. The sampling procedures aimed to gather a mix of domestic (*n* = 280) and imported produce samples (*n* = 63) from conventional cultivation (*n* = 268) and soil-less (e.g., hydroponic) produce (*n* = 75). Each sample was inspected for visible dirt and spoilage and transported to the analysis laboratory at the United Arab Emirates University in Al Ain in a cooling box. This method ensured the sample’s integrity until it reached the lab for same-day processing.

### 2.2. Bacterial Isolation

For microbiological analysis, 25 g of each sample were mixed with 225 mL of buffered peptone water (Oxoid, Basingstoke, UK) and homogenized using a paddle blender (BagMixer^®^ 400 P, Interscience, Saint-Nom-la-Breteche, France) for 120 s. The procedure followed the International Organization for Standardization (ISO 6888-1) guidelines [11]. To isolate *Staphylococcus* spp., three decimal dilutions were prepared, and 1 mL from each dilution was spread onto three Baird–Parker agar plates, which were enriched with egg yolk emulsion and 1% potassium tellurite (Oxoid, UK). These plates were incubated at 37 °C for 48 h [11]. Typical suspected colonies were counted, and representative selections (up to five colonies) were re-streaked for purity on the surface of nutrient agar plates (Oxoid, UK). Isolates were kept on glycerol stock at −80 °C for further confirmation and identification. While CoNS can grow on Baird–Parker agar, they typically do not produce the characteristic black colonies with clear zones indicative of *S. aureus*. CoNS colonies may appear small, gray, or white without distinct zones of clearing [5,11].

### 2.3. Characterization of Staphylococcus spp.

Up to five suspected colonies (displaying black to gray coloration, with or without a clear zone around) per sample were confirmed to species level using Matrix-Assisted Laser Desorption Ionization-Time of Flight Mass Spectrometry (MALDI-TOF MS) with the Autobio ms1000 instrument (Autobio Diagnostics, Zhengzhou, China). The 96-spot target plate was calibrated daily, and a well-isolated colony was spread onto the target spot and admixed with 1 μL of α-cyano-4-hydroxycinnamic acid, CHCA, according to manufacturer-recommended procedures (Autobio Diagnostics, China). The Autobio ms1000 results were reported as “reliable identification to species level” for scores between 9.000 and 10.000 [12].

Additionally, all MALDI-TOF-confirmed isolates were further analyzed using a triplex PCR assay to differentiate *S. aureus* from coagulase-negative *Staphylococci* and to determine methicillin resistance [13]. Genomic DNA was extracted using the commercially available DNA Purification Kit (Wizard^®^; Promega, Madison, WA, USA). The PCR method targeted three specific genes: *mecA*, indicating methicillin resistance; *nuc*, specific to *S. aureus*, encoding the thermonuclease gene; and a genus-specific 16S rRNA sequence, serving as an internal control for *staphylococcal* DNA [13]. A NanoDrop spectrophotometer (Thermo Scientific, Waltham, MA, USA) and a Quantus fluorometer (Promega, USA) were used to evaluate the quality of the gDNA.

### 2.4. Testing the Susceptibility of Staphylococcus spp. Isolates to Antimicrobials

The minimum inhibitory concentration (MIC) testing for *Staphylococcus* spp. isolates was conducted using the VITEK-2 system (bioMérieux, Craponne, France). The testing utilized VITEK 2 AST-P592 cards (bioMérieux, France) specifically designed for *Staphylococci* susceptibility testing, following the manufacturer’s guidelines. The antimicrobial agents tested included benzylpenicillin (concentrations: 0.125 mg/L; 0.25 mg/L; 1 mg/L), cefoxitin screen test (negative/positive), oxacillin (concentrations: 0.5 mg/L; 1 mg/L; 2 mg/L), gentamicin high level (concentration: 500 mg/L), ciprofloxacin (concentrations: 1 mg/L; 2 mg/L; 4 mg/L), moxifloxacin (concentrations: 0.25 mg/L; 2 mg/L; 8 mg/L), inducible clindamycin resistance (negative/positive), clindamycin (concentrations: 0 mg/L, 5 mg/L; 1 mg/L; 2 mg/L), linezolid (concentrations: 0.5 mg/L; 1 mg/L; 2 mg/L), Teicoplanin (concentrations: 1 mg/L; 4 mg/L; 8 mg/L), vancomycin (concentrations: 1 mg/L; 2 mg/L; 4 mg/L; 8 mg/L; 16 mg/L), tetracycline (concentrations: 0.5 mg/L; 1 mg/L; 2 mg/L), tigecycline (concentrations: 0.25 mg/L; 0.5 mg/L; 1 mg/L), rifampicin (concentrations: 0.25 mg/L; 0.5 mg/L; 2 mg/L), and trimethoprim/sulfamethoxazole (concentrations: 8/152 mg/L; 16/302 mg/L; 32/608 mg/L). Isolates were designated as resistant according to the Clinical and Laboratory Standards Institute guidelines [14]. Multidrug-resistant (MDR) isolates are defined as those exhibiting resistance to at least three different classes of antibiotics from the panel used [15].

### 2.5. Whole Genome Sequencing and Prediction of Resistome and Virulome

WGS was performed for a selection of isolates (*n* = 10), chosen based on their oxacillin susceptibility and/or cefoxitin screen test, and included those exhibiting an MDR (resistant to at least three classes of antimicrobials) profile. The sequencing was completed by Novogene Company (Hong Kong, China) using Illumina HiSeq 2500 (Illumina, San Diego, CA, USA) and paired-end 150 bp reads with 100× coverage. Genome sequences were reassembled using SPAdes software (version 3.15.5) with default parameters [16]. Bioinformatics techniques were applied using the Solu online platform (Solu Healthcare Inc., Helsinki, Finland; https://platform.solu.bio/ (accessed on 15 May 2024)). The Solu platform provided a batch analysis of the species identity, multi-locus sequence type (MLST), antimicrobial resistance genes and mutations, and virulence factors. A gene identity threshold was set at ≥95% and a cut-off value of 60% of the sequence coverage [17]. The raw reads were submitted to the National Library of Medicine (NCBI) under BioProject ID: PRJNA1119919.

### 2.6. Statistical Analysis 

Statistical differences (*p* < 0.05) in *Staphylococci* detection among various types of salad items were assessed using the non-parametric Mann–Whitney test for pairwise comparisons between two independent variables and the Kruskal–Wallis test for multiple comparisons involving more than two independent variables. All statistical analyses were conducted using GraphPad Prism (version 6.0; GraphPad Software, Inc., La Jolla, CA, USA).

## 3. Results

### 3.1. Frequency of Staphylococcus spp.

**Figure 2:**
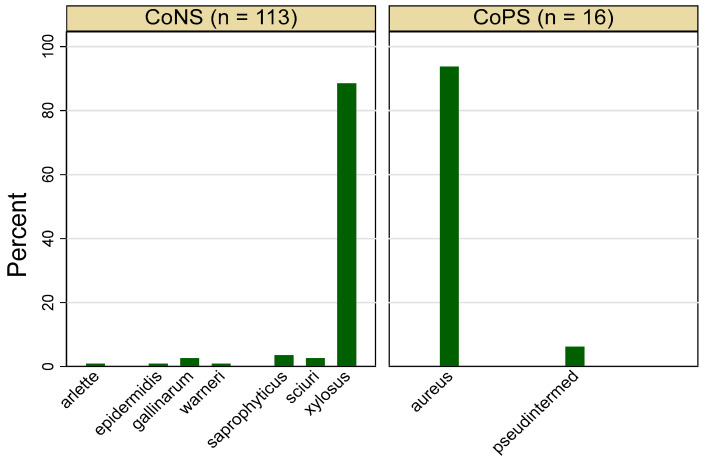
Distribution of coagulase-positive (CoPS) and coagulase-negative (CoNS) *Staphylococcus* spp. among positive isolates (*n* = 129) recovered from different salad items sampled from markets in the United Arab Emirates.

Figure 1 shows that *Staphylococcus* spp. were detected in 37.6% (129/343) of the tested salad items, with a maximum detection rate of 60% (21/35) in arugula samples and a minimum of 19.3% (6/31) in iceberg lettuce. As indicated in Figure 2, nine *Staphylococcus* species were identified among the tested salad items. Coagulase-negative *Staphylococci* (CoNS) dominated, comprising 87.6% (113/129) of the *Staphylococcus* spp., with *S. xylosus* being the most prevalent at 89.4% (101/113) (Figure 2). In contrast, coagulase-positive *Staphylococci* (CoPS) accounted for only 12.4% (16/129) of the *Staphylococcus* spp., with *S. aureus* detected in just 4.6% (14/343) of the overall tested salad vegetable samples (Figure 2), with an average count of 1.7 log10 CFU/g.

### 3.2. Screening of Antimicrobial Phenotypic Resistance

A total of 62 non-duplicate isolates were screened for antimicrobial resistance (Table 1). These isolates included the two dominant species recovered from the salad items tested in this study, namely *S. xylosus* (*n* = 45) and *S. aureus* (*n* = 10), as well as other *Staphylococci* species (*n* = 7). Among the 45 *S. xylosus* isolates, 16 (35.6%) were resistant to eight (50.0%) of the 16 tested antibiotics, of which one was an MDR isolate (Table 1). It is worth highlighting that 13.3% (6/45) of *S. xylosus* isolates were positive in the cefoxitin screen test, and 6.6% (3/45) were non-susceptible to oxacillin (Table 1). Of the characterized *S. aureus* isolates, only one (STAP_10) was positive in the cefoxitin screen test and was also oxacillin-resistant (Table 2). Additionally, 40.0% (4/10) and 20.0% (2/10) of the *S. aureus* isolates were non-susceptible to trimethoprim/sulfamethoxazole and ciprofloxacin, respectively (Table 1). Four isolates (57.1%) from four different species were resistant to erythromycin, and only two isolates, one each of *S. xylosus* and *S. aureus*, were identified as multidrug-resistant (MDR) (Table 1).

### 3.3. Genomic Insight on Antimicrobial Resistance Determinants

**Figure 3:**
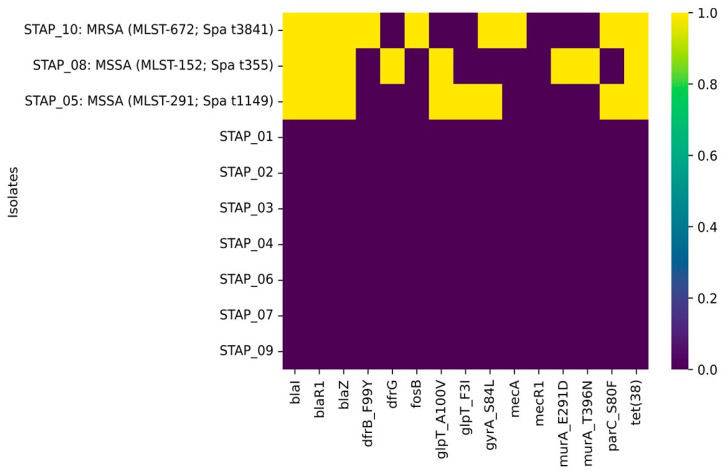
Antimicrobial resistance genetic determinants among whole-genome sequenced *staphylococci* isolates recovered from different salad items sampled from markets in the United Arab Emirates. The color in the heatmap indicates whether a gene is present (yellow color) or absent (purple color) in an isolate.

As indicated in Table 2, WGS was performed on ten isolates, chosen primarily based on their diverse antimicrobial resistance profiles. The WGS analysis of six *S. xylosus* and one *S. warneri* did not identify any known antimicrobial resistance determinants (e.g., genes or mutations) in the genome sequences of such isolates. Among the three *S. aureus* isolates, the isolate STAP_10, obtained from an imported dill sample (Table 2), was identified as methicillin-resistant (MRSA) due to the presence of the *mecA* gene, as confirmed by WGS analysis (Figure 3). This was in keeping with the triplex PCR results that also detected the *mecA* gene in this isolate. There was concordance between genotypic and phenotypic screening for this isolate, as it tested positive in the cefoxitin screen test and was resistant to oxacillin (Table 2). This isolate belonged to multi-locus sequence type (MLST) ST-672 and spa type t3841 (Figure 3). The other *S. aureus* isolates were methicillin-sensitive (MSSA), tested negative for the cefoxitin screen test (Table 2), and belonged to ST-152, spa type t355 (STAP_08), and ST-291, spa type t1149 (STAP_05) (Figure 3). 

As shown in Figure 3, all three *S. aureus* isolates harbored genes indirectly involved in regulating beta-lactamase expression (*blaI* and *blaR1*). Additionally, the three isolates carried the *blaZ* gene, which directly hydrolyzes the beta-lactam ring. Two of the three isolates (STAP_10 and STAP_05) harbored two mutations known to have been reported to confer resistance to fluoroquinolones by reducing drug binding affinity: *gyrA*_*S84L* in the DNA gyrase gene and *parC*_*S80F* in the topoisomerase IV gene. Trimethoprim resistance determinants were also revealed in two isolates: *dfrB*_*F99Y* in STAP_08 and *dfrG* in STAP_10 (Figure 3). Several fosfomycin resistance determinants were found among the three *S. aureus* isolates, including mutations in the glycerol-3-phosphate transporter (glpT_A100V and glpT_F3I) and in the enzyme involved in peptidoglycan synthesis (murA_E291D and murA_T396N) (Figure 3).

### 3.4. Genomic Insight on Toxin-Related Factors

**Figure 4:**
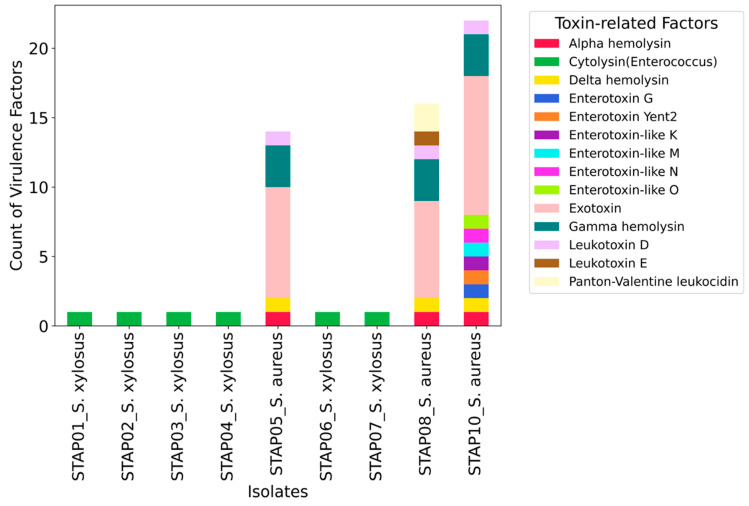
Distribution of toxin-related factors across whole-genome sequenced *Staphylococci* isolates recovered from different salad items sampled from markets in the United Arab Emirates. Each bar represents an isolate, and the different colors within each bar correspond to different toxin-related factors. The height of each colored segment indicates the count of specific toxin-related genes present in that isolate.

Based on WGS analysis, we identified the cytolysin gene (*cylR2*) as the only toxin-related factor in *S. xylosus*; however, this gene was not present in the *S. aureus* isolates characterized in this study (Figure 4). As shown in Figure 4, *S. aureus* isolates harbored a wide range of toxin-related factors. The methicillin-resistant isolate (STAP_10) possessed 22 toxin-related genes, compared to 16 and 14 genes in isolates STAP_08 and STAP_05, respectively (Figure 4). The methicillin-resistant isolate (STAP_10) contained several enterotoxin-related genes not present in the other MSSA isolates (STAP_08 and STAP_05), including seg (enterotoxin G), *yent2* (enterotoxin Yent2), *selk* (enterotoxin-like K), *selm* (enterotoxin-like M), *seln* (enterotoxin-like N), and *selo* (enterotoxin-like O). Notably, the WGS analysis of the MSSA isolate STAP_08 revealed the presence of Panton-Valentine Leukocidin (*LukSF/PVL*) (Figure 4).

## 4. Discussion

This study investigated the presence of *Staphylococcus* spp. in various salad items sampled from markets in the United Arab Emirates, providing the first comprehensive analysis of the biodiversity of these species in fresh produce within the region. Consistent with our study, researchers have demonstrated the presence of both CoPS and CoNS in retail vegetables and fruits in various countries [9,18]. Moreover, according to two recent surveys, *S. xylosus* was among the top five most commonly detected CoNS species in salad items [18,19]. Comparable to our findings, a study in Ghana reported the presence of *S. xylosus* in 91.6% of food samples from two cities [18]. *S. xylosus* is ubiquitous, found in various niches, and persists in soil and on surfaces. Its ability to form biofilms and adapt to different environments likely explains its wide presence as a commensal, including its presence in soil and agricultural ecosystems [20]. Although this species is typically regarded as nonpathogenic, a few strains have been associated with opportunistic infections in animals and humans [20,21]. Therefore, while most strains are harmless commensals, some have the potential to be hazardous. This underscores the versatility of this species and the importance of understanding its characteristics in the fresh produce supply chain.

Despite the evident detection of CoNS in animal and plant-based foods, standards and food safety regulations do not require monitoring of CoNS, focusing solely on the presence of *S. aureus* [22]. Some reports have indicated that *S. aureus* is the major pathogen in ready-to-eat salads due to contamination during food preparation and processing by workers [23]. According to the present study, the prevalence of *S. aureus* in salad vegetables sampled from retail outlets in the UAE was 4.6% (16/343). This prevalence is considerably lower than the 10% pooled prevalence found in a meta-analysis of the global prevalence of *S. aureus* in vegetable items [24]. Nevertheless, the risk to consumers from *S. aureus* is not only related to its prevalence but also to the consumption rate of salads and the potential for *S. aureus* growth in these foods [25]. The interaction of these factors needs to be assessed further to estimate the probability of *S. aureus* causing foodborne illness.

Methicillin-resistant *Staphylococci* are defined as carrying the *mecA* gene (or, less commonly, *mecB* or *mecC*) or phenotypically showing resistance to oxacillin or cefoxitin [26]. Our results indicate the phenotypic detection of only one methicillin-resistant *S. aureus* isolate, but more resistance was revealed among *S. xylosus*, with 13.3% positive in the cefoxitin screen test and 6.6% non-susceptible to oxacillin. It should be emphasized that cefoxitin and oxacillin susceptibility testing are equally important, as one method may detect resistant isolates that the other cannot [27]. Either a phenotypic or genotypic-resistant result would require reporting isolates as methicillin-resistant [28].

The frequency of potential methicillin resistance among *S. xylosus*, the major CoNS species found in salad items tested in our study, is considerably lower compared to those reported in the literature. A recent study in Poland found that 78.8% of CoNS from ready-to-eat food samples, including salads, were resistant to at least one tested antibiotic, and 36.5% were phenotypically methicillin-resistant [22]. In Greece, 41.7% of CoNS isolated from ready-to-eat foods were methicillin-resistant isolates [29]. Thus, although CoNS species, such as *S. xylosus*, are typically considered commensal contaminants, this group of *Staphylococci* might still carry potential resistance to clinically relevant antibiotics and should not be overlooked in One Health surveillance of antimicrobial resistance at the human-food interface.

According to the records deposited into the public databases for molecular typing and microbial genome diversity (PubMLST), the first reported isolate belonged to ST-672, which we identified as well in the only MRSA isolate confirmed in the present study, was identified in India in a human clinical isolate (from invasive blood infection) in 2003, which was also MRSA (PubMLST database, https://pubmlst.org/organisms/staphylococcus-aureus; accessed on 31 May 2024). Since then, all identified ST-672 isolates globally (*n* = 26, recorded isolates to the PubMLST database) have also been MRSA and mostly isolated from hospital or community-acquired infections, as well as from animal (dairy cows) sources (PubMLST database, https://pubmlst.org/organisms/staphylococcus-aureus; accessed on 31 May 2024). These results indicate that ST-672 is globally distributed and has been proven to be involved in human illnesses. In the UAE, ST-672 has not been reported in human MRSA clinical isolates, while *S. aureus* belonging to ST-152 (that we found in one MSSA (isolate STAP_08) in the present study) has been reported before in clinical isolates in the UAE [26].

Although the current study is not the first to identify MRSA in vegetables [9,30], to the best of our knowledge, this is the first reported MRSA in the food chain in the UAE. Moreover, given that the sample carrying MRSA was imported, this work further highlights the possible intercontinental transmission of resistant organisms, such as MRSA, via internationally traded vegetables [30]. The detection of MRSA in retail fresh products illustrates the complexity of contamination pathways, necessitating stringent hygiene and monitoring practices. The risk associated with *Staphylococci* is due to their pathogenic properties and capacity to acquire mobile genetic elements encoding antimicrobial resistance genes via horizontal gene transfer [31,32]. Our results highlight the importance of vigilant sampling and screening of salad vegetables to monitor and control the spread of antimicrobial resistance. Given that the prevalence of *S. aureus* based on the current study findings was low (4.6%) and only one isolate was found to be MRSA, the immediate risk appears minimal. However, ongoing surveillance remains crucial to prevent the potential emergence or spread of unwanted resistance mechanisms and ensure food safety [33].

In *Staphylococcus* spp., toxin-related factors are crucial for their virulence as they enable the bacteria to evade the host immune system, cause tissue damage, and contribute to the severity of infections [34,35]. Cytolysins, presented in the *S. xylosus* isolates characterized in this study, are potent toxins that can lyse host cells, leading to more severe infections and complications [34]. The hypothesized transfer of this gene between different bacterial species, apparently *Enterococcus*, as pointed out from the WGS analysis, also suggests a role in enhancing bacterial fitness and adaptability in diverse environments [34]. This highlights the importance of monitoring horizontal gene transfer in microbial populations in the agriculture web at the human-food interface.

The present study also reports the first characterization of a *PVL*-positive and *mecA*-negative *S. aureus* strain in the UAE food chain. The biological relevance of this finding lies in the *PVL* toxin’s ability to destroy white blood cells, which can lead to severe skin infections and, in rare cases, necrotizing pneumonia [35]. While *PVL* is a significant virulence factor, it has not been commonly associated with foodborne transmission [36]. A high occurrence of *PVL*-positive isolates has been reported in the region. Given that this toxin was found in only one isolate, the immediate public health risk is questionable. However, it is worth noting here that the percentage of *PVL* positivity in *staphylococcus* isolates in the Gulf Cooperation Council region, including the UAE, is uniquely high compared to other geographic regions [37]. Hence, continued monitoring remains essential to detect any changes in prevalence and the implications for potential introductions of *PVL*-positive/*mecA*-negative *Staphylococci* strains via the food chain.

## 5. Conclusions 

This study offers the first comprehensive analysis of *Staphylococcus* spp. biodiversity in fresh produce from UAE markets, revealing important findings on the prevalence, antimicrobial resistance, and toxin-related factors of these bacteria. The detection of MRSA in imported salad items highlights the potential for trade-related transmission of antimicrobial-resistant bacteria, an anticipated matter that seems unavoidable yet warrants more vigilant monitoring. Our findings underscore the necessity of including coagulase-negative *Staphylococci* (CoNS) in food safety standards and ongoing surveillance. Despite being primarily commensals, CoNS, such as *S. xylosus*, which may dominate certain food items like salad vegetables, can acquire and potentially transfer antimicrobial resistance due to their widespread presence in the environment. Future research should expand antimicrobial surveillance, conduct longitudinal and mechanistic studies, perform risk assessments, and advocate for comprehensive food safety policies to protect public health from antimicrobial-resistant and toxin-producing *Staphylococcus* spp. in fresh produce.

## Figures and Tables

**Table 1:** Antimicrobial resistance characterization of *Staphylococcus xylosus*, *Staphylococcus aureus*, and other *Staphylococcus* spp. isolates recovered from different salad items sampled from markets in the United Arab Emirates.

Antimicrobials	*Staphylococcus xylosus* (*n* = 45)	*Staphylococcus aureus* (*n* = 10)	Other *Staphylococci* (*n* = 7)
	Resistant No. (%)	Resistant No. (%)	Resistant No. (Species)
Benzylpenicillin	1 (2.2)	1 (10.0)	2 (*S. warneri*, *S. saprophyticus*)
Oxacillin	3 (6.6)	1 (10.0)	2 (*S. warneri*, *S. gallinarum*)
Gentamicin	0 (0.0)	0 (0.0)	—
Ciprofloxacin	0 (0.0)	2 (20.0)	—
Moxifloxacin	0 (0.0)	0 (0.0)	—
Erythromycin	2 (4.4)	0 (0.0)	4 (*S. arlette*, *S. warneri*, *S. saprophyticus*, *S. gallinarum*)
Clindamycin	1 (2.2)	0 (0.0)	2 (*S. warneri*, *S. saprophyticus*)
Linezolid	0 (0.0)	0 (0.0)	—
Teicoplanin	1 (2.2)	0 (0.0)	1 (*S. warneri*)
Vancomycin	1 (2.2)	0 (0.0)	1 (*S. warneri*)
Tetracycline	0 (0.0)	0 (0.0)	1 (*S. warneri*)
Tigecycline	0 (0.0)	0 (0.0)	1 (*S. warneri*)
Rifampicin	1 (2.2)	0 (0.0)	1 (*S. warneri*)
Trimethoprim/Sulfamethoxazole	0 (0.0)	4 (40.0)	1 (*S. warneri*)
	**Positive No. (%)**	**Positive No. (%)**	**Positive No. (%)**
Cefoxitin Screen test	6 (13.3)	1 (10.0)	2 (*S. warneri*, *S. gallinarum*)
Inducible Clindamycin resistance	0 (0.0)	0 (0.0)	1 (*S. saprophyticus*)
	**No. (%)**	**No. (%)**	**No. (%)**
Multidrug-resistant	1 (2.2)	1 (10.0)	2 (*S. warneri*, *S. gallinarum*)

**Table 2:** Phenotypic antimicrobial resistance pattern of ten whole-genome sequenced *Staphylococci* isolates recovered from different salad items sampled from markets in the United Arab Emirates (UAE).

Isolate	Species	Sample	Origin	Resistance Phenotypic Profile
STAP_01	*Staphylococcus xylosus*	Spinach	UAE	Cefoxitin screen test + ve
STAP_02	*Staphylococcus xylosus*	Arugula	UAE	Cefoxitin screen test + ve Benzylpenicillin Oxacillin Erythromycin
STAP_03	*Staphylococcus xylosus*	Bagged mix	UAE	Cefoxitin screen test + ve Oxacillin
STAP_04	*Staphylococcus xylosus*	Iceberg lettuce	Spain	Cefoxitin screen test + ve
STAP_05	*Staphylococcus aureus*	Parsley	UAE	Cefoxitin screen test − ve Ciprofloxacin
STAP_06	*Staphylococcus xylosus*	Roman lettuce	Jordan	Cefoxitin screen test + ve Oxacillin Erythromycin Clindamycin Teicoplanin Vancomycin Rifampicin
STAP_07	*Staphylococcus xylosus*	Cabbage	UAE	Cefoxitin screen test − ve
STAP_08	*Staphylococcus aureus*	Arugula	Egypt	Cefoxitin screen test − ve Trimethoprim/sulfamethoxazole
STAP_09	*Staphylococcus warneri*	Spinach	Egypt	Cefoxitin screen test + ve Benzylpenicillin Oxacillin Erythromycin Clindamycin Teicoplanin Vancomycin Tetracycline Tigecycline Rifampicin Trimethoprim/sulfamethoxazole
STAP_10	*Staphylococcus aureus*	Dill	Egypt	Cefoxitin screen test + ve Benzylpenicillin Oxacillin Ciprofloxacin

## Data Availability

The original contributions presented in the study are included in the article; further inquiries can be directed to the corresponding author.
